# Loss of Sbds in zebrafish leads to neutropenia and pancreas and liver atrophy

**DOI:** 10.1172/jci.insight.134309

**Published:** 2020-09-03

**Authors:** Usua Oyarbide, Arish N. Shah, Wilmer Amaya-Mejia, Matthew Snyderman, Margaret J. Kell, Daniela S. Allende, Eliezer Calo, Jacek Topczewski, Seth J. Corey

**Affiliations:** 1 Departments of Pediatrics, Immunology, and Human and Molecular Genetics, Children’s Hospital of Richmond and Massey Cancer Center at Virginia Commonwealth University, Richmond, Virginia, USA.; 2 Department of Pediatrics, Stanley Manne Children’s Research Institute, Northwestern University School of Medicine, Chicago, Illinois, USA.; 3 Departments of Pediatrics, Cancer Biology, and Translational Hematology and Oncology Research, Cleveland Clinic, Cleveland, Ohio, USA.; 4 Department of Biology and David H. Koch Institute for Integrative Cancer Research, Massachusetts Institute of Technology, Cambridge, Massachusetts, USA.; 5 Department of Pathology, Cleveland Clinic, Cleveland, Ohio, USA.; 6 Department of Biochemistry and Molecular Biology, Medical University of Lublin, Lublin, Poland.

**Keywords:** Gastroenterology, Hematology, Embryonic development, Genetic diseases, P53

## Abstract

Shwachman-Diamond syndrome (SDS) is characterized by exocrine pancreatic insufficiency, neutropenia, and skeletal abnormalities. Biallelic mutations in *SBDS*, which encodes a ribosome maturation factor, are found in 90% of SDS cases. *Sbds^–/–^* mice are embryonic lethal. Using CRISPR/Cas9 editing, we created *sbds-*deficient zebrafish strains. Sbds protein levels progressively decreased and became undetectable at 10 days postfertilization (dpf). Polysome analysis revealed decreased 80S ribosomes. Homozygous mutant fish developed normally until 15 dpf. Mutant fish subsequently had stunted growth and showed signs of atrophy in pancreas, liver, and intestine. In addition, neutropenia occurred by 5 dpf. Upregulation of *tp53* mRNA did not occur until 10 dpf, and inhibition of proliferation correlated with death by 21 dpf. Transcriptome analysis showed tp53 activation through upregulation of genes involved in cell cycle arrest, *cdkn1a* and *ccng1*, and apoptosis, *puma* and *mdm2*. However, elimination of Tp53 function did not prevent lethality. Because of growth retardation and atrophy of intestinal epithelia, we studied the effects of starvation on WT fish. Starved WT fish showed intestinal atrophy, zymogen granule loss, and tp53 upregulation — similar to the mutant phenotype. In addition, there was reduction in neutral lipid storage and ribosomal protein amount, similar to the mutant phenotype. Thus, loss of Sbds in zebrafish phenocopies much of the human disease and is associated with growth arrest and tissue atrophy, particularly of the gastrointestinal system, at the larval stage. A variety of stress responses, some associated with Tp53, contribute to pathophysiology of SDS.

## Abstract 2

Loss of ribosome maturation factor sbds in the zebrafish phenocopies human Shwachman-Diamond syndrome and is associated with p53 activation, but lethality cannot be rescued by p53 mutation.

## Introduction

Originally described in 2 reports in 1964, Shwachman-Diamond syndrome (SDS, OMIM 260400) is classically characterized by exocrine pancreatic insufficiency, neutropenia, and skeletal abnormalities. Exocrine pancreatic insufficiency occurs early in childhood, intermittently requires enzyme supplementation, and is characterized by fatty replacement of the pancreas with sparing of the beta islet cells and ductal architecture. Fatty infiltration also occurs amid the hypocellular bone marrow. Clinical findings are heterogeneous and may wax and wane (1). Elevated transaminases, steatorrhea, failure to thrive, short stature, anemia, thrombocytopenia, infections, and rib cupping are frequently observed (2, 3). Better clinical recognition and next-generation sequencing are identifying affected adults (4, 5).

Approximately 90% of SDS cases are caused by biallelic mutations in the SBDS ribosome maturation factor (*SBDS*) gene (6). The most common *SBDS* mutations are located in exon 2 and lead to protein truncation after introduction of a stop codon (K62X) or disruption of a donor splice and a frameshift (C83fs). C83fs mutation produces reduced expression of full-length protein (7). While a few patients are homozygous for the splice donor mutation, homozygous mutants for K62X have not been described, suggesting that complete loss of SBDS function is lethal (6–8). At least 90% of patients with SDS, carrying *SBDS* mutations, have 1 of these 2 mutations (6, 8). Studies in yeast, mice, and human cells have shown a role of SBDS in 60S ribosome maturation. SBDS interacts with the GTPase elongation factor-like 1 to dissociate eukaryotic initiation factor 6 (eIF6) from the cytoplasmic 60S ribosomal subunit, allowing assembly of 80S ribosomes ready for translation (6, 9–12). In addition, SBDS deficiency affects different cellular processes, including cell viability and/or proliferation (13, 14), chemotaxis (15), cellular stress response (16), Fas ligand–induced apoptosis (17), and regulation of ROS levels (18).

Recently, mutations in genes associated with ribosome biogenesis and function, *DNAJC21* (19, 20) (involved in late cytoplasmic 60S ribosomal subunit maturation) (21) and *EFL1* (a partner to SBDS promoting the assembly of the 40S and 60S subunits) (22, 23), and *SRP54* (binds to the nascent polypeptide as it emerges from the 80S ribosome) (24, 25), have been described in patients with SDS-like conditions. Numerous blood and nonhematological disorders arise from genetic variants, affecting ribosome structure and function (26). The mechanisms underlying these ribosomopathies are not well understood. Several studies suggest activation of the Tp53 pathway in the pathogenesis of Diamond-Blackfan anemia, another ribosomopathy, in both mouse and zebrafish models (27–29). Treacher Collins syndrome, which does not affect the blood system, results from mutations in *TCOF1*, involved in production of rRNA, or in *POLR1C* and *POLR1D* (30), encoding subunits of RNA polymerases I and III involved in ribosome biogenesis. Zebrafish *polr1c* mutants showed an upregulated Tp53 pathway (31). However, morpholinos may themselves trigger Tp53 activation (32), and other ribosomopathies suggest that Tp53-independent pathways also contribute to bone marrow failure (33). Very little is known about how SBDS affects development and tissue maintenance of the gastrointestinal system.

Understanding the pathophysiology of SDS has been difficult because of its rarity, phenotype variability, multiple organ involvement, and paucity of organismal models. Ablation of SBDS in mice produces early lethality (E6.5) with failure in epiblast development (34). Zebrafish with morpholino-mediated knockdown of *sbds* survive for only the first few days postfertilization and recapitulate organ abnormalities observed in patients with SDS (35, 36). Using CRISPR/Cas9 genome editing, we generated zebrafish strains with indel mutations in *sbds* that result in loss of normal protein. These zebrafish recapitulate the hallmarks of SDS: neutropenia, pancreatic atrophy, and short stature (length). Analysis of differential gene expression revealed dynamic activation of Tp53-associated gene targets, but Tp53 loss of function itself did not completely rescue the phenotype. Moreover, when WT fish underwent starvation at the same developmental stage, they showed stunting and Tp53 pathway activation similar to the *sbds*-mutant fish.

## Results

### Generation and characterization of sbds-mutant alleles in zebrafish.

**Figure 1:**
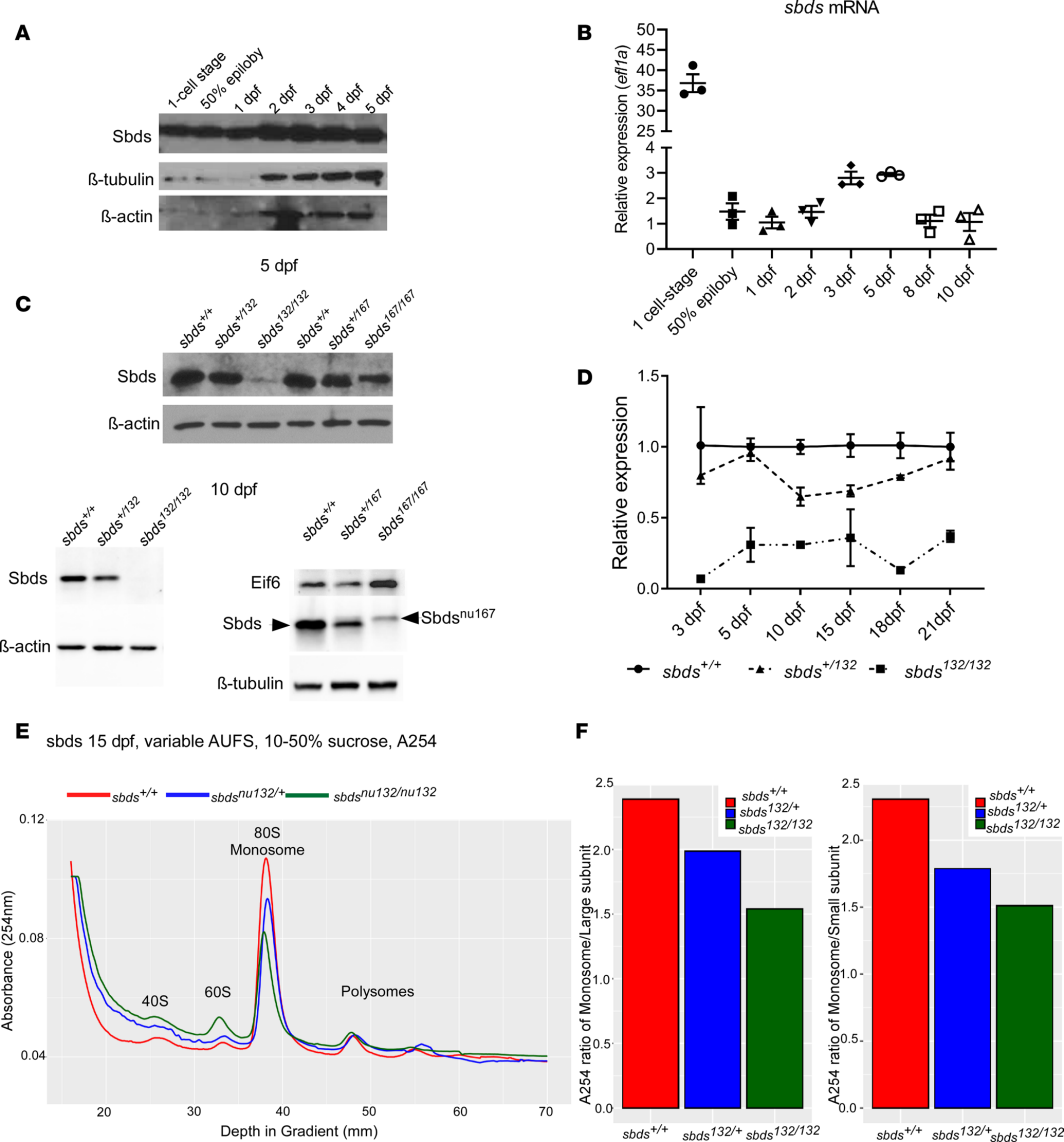
Sbds and *sbds* expression during development. (**A**) Western blot of lysates from 25–30 pooled WT embryos showing Sbds expression at different stages in WT zebrafish. (**B**) *Sbds* expression profile in WT zebrafish the first 10 dpf with *eifl1a* as housekeeping gene. Gene expression normalized to 1 dpf and *efl1a*. (**C**) Western blotting showing Sbds expression at 5 and 10 dpf. (**D**) *Sbds* mRNA expression at different ages for WT, heterozygous, and homozygous mutants for the *sbds^nu132^* mutation. Expression is normalized against β-actin. (**E**) Polysome profile of 15 dpf larvae shows an increase of 60S and lower 80S peaks on sucrose gradient in *sbds* mutants and heterozygotes compared with WT siblings. (**F**) Ratio of monosome with respect to 40S and 60S subunits. Mutants and heterozygotes showed a lower ratio compared with WT siblings. AUFS, absorbance units full scale.

The human SBDS protein has a mass of approximately 27 kDa, and its primary amino acid sequence is highly conserved among vertebrates (Supplemental Figure 1A; supplemental material available online with this article; https://doi.org/10.1172/jci.insight.134309DS1). The human SBDS is 97% identical to murine and 86% to zebrafish orthologs. Through CRISPR/Cas9 editing, 7 indel mutations were generated in 3 founders (Supplemental Table 1). We focused on 2 mutations to create stable lines: *sbds^nu132^*, a 7 bp deletion that caused a protein truncation, and *sbds^nu167^*, a 21 bp insertion that resulted in an in-frame insertion of 7 amino acids. To study maternal deposition, we analyzed gene and protein expression at selected time points postfertilization. We observed a significant amount of *sbds* mRNA and protein expression at the 1-cell stage (Figure 1, A and B). Western blotting analysis revealed reduced expression of full-length Sbds at 3 days after fertilization (dpf) (data not shown), with a progressive decline from 5 dpf *sbds^nu132^* mutants to its absence at 10 dpf (Figure 1C). Using reverse transcription quantitative PCR (RT-qPCR) gene expression analysis of embryos from an incross of *sbds^nu132/+^*, we found a significant downregulation of *sbds* at all stages (Figure 1D and Supplemental Figure 2A). Moreover, *sbds^nu132/+^* also showed a marked downregulation compared with their WT siblings at all stages and showed an intermediate protein quantity between WT and the *sbds^nu132^* mutants. As predicted, in lysates obtained from *sbds^nu167^* mutants, we detected a protein of slightly higher molecular mass. The corresponding band was fainter than the WT Sbds (Figure 1C). mRNA levels of *sbds* in *sbds^nu167/nu167^* were also downregulated at 10 dpf (Supplemental Figure 2B). Polysome profiling showed a reduction in the 80S ribosome in *sbds^nu132/nu132^* and *sbds^nu132/+^* when compared with their WT siblings (Figure 1E). Furthermore, the ratio of the monosome peak to the large or small subunits was reduced strongly in mutants and intermediately in heterozygous larvae (Figure 1F).

### Human SDS is phenocopied by sbds^nu132/nu132^ mutants.

**Figure 2:**
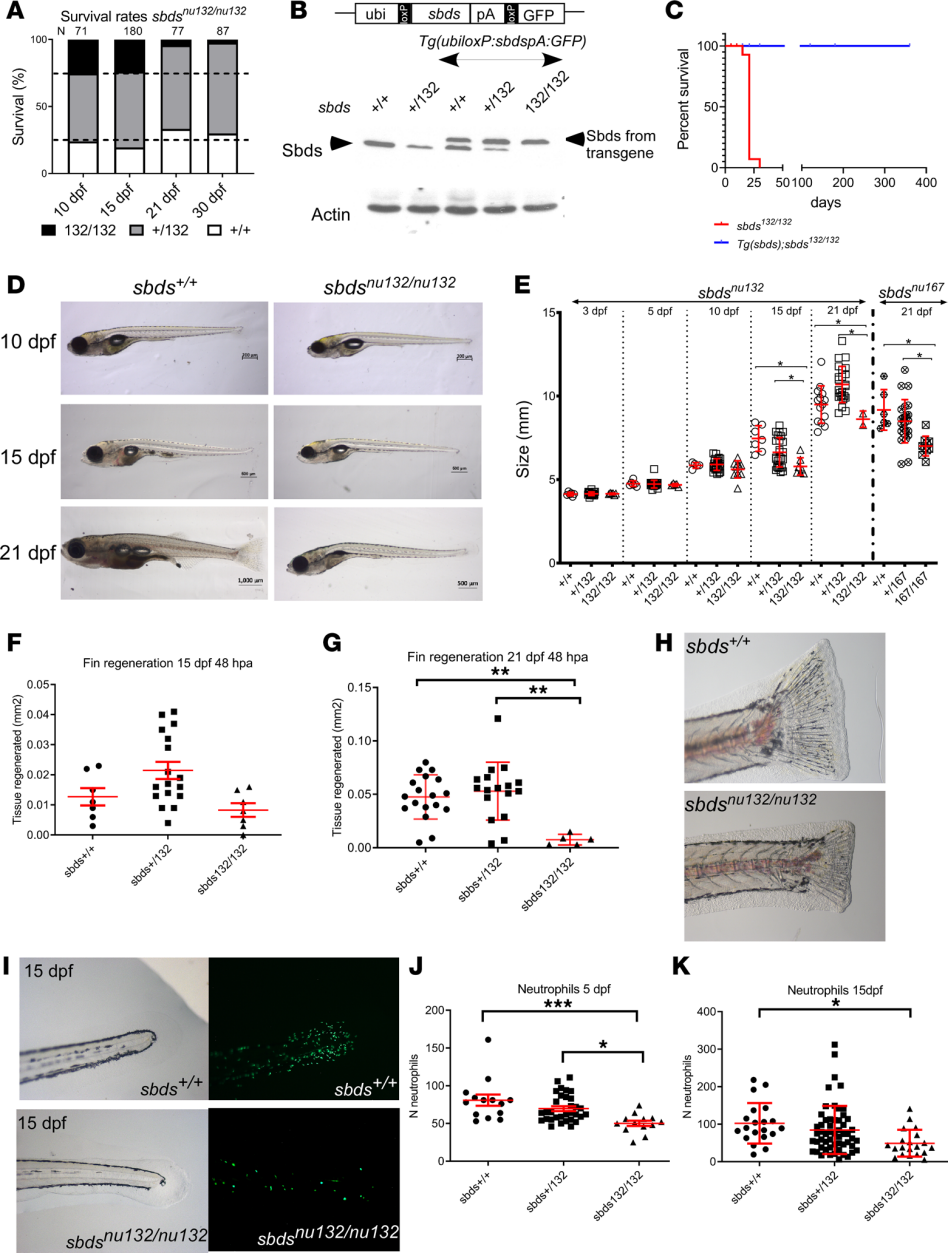
Sbds mutation leads to a defect in growth and fin regeneration and neutropenia. (**A**) Survival rates for siblings of *sbds^nu132^*. (**B**) Rescue of *sbds* mutants: Western blot showing Sbds from fins of adult fish with the indicated different genotypes. Note that the transgenic line expresses Sbds of a slightly bigger mass, due to an inadvertently introduced initiation codon upstream of the coding sequence. We took advantage of this 23–amino acid tag to distinguish between endogenous and exogenously introduced Sbds. Actin is shown as a control for protein loading. (**C**) Survival analysis, which demonstrates the rescue of *sbds^nu132/nu132^* by the transgenic line *Tg(ubiloxP:sbdsloxP:GFP)* *N* = 50; *sbds^nu132/nu132^*. (**D**) Images of 10, 15, and 21 dpf larvae from the same clutch: *sbds^+/+^* and *sbds^nu132/nu132^*. Scale bars: 200 μm (10 dpf), 500 μm (15 dpf), 1000 μm (*sbds^+/+^* 21 dpf), and 500 μm (*sbds^nu132/nu132^* 21 dpf). (**E**) Size variability of mutants (*sbds^nu132^* and *sbds^nu167^*) in the first 21 dpf. Fin regeneration 48 hours after amputation (hpa) in fish that are (**F**) 15 dpf (*N* = 30) and (**G**) 21 dpf, which shows less regeneration (*N* = 42). (**H**) Representative images of the fins 48 hpa in 21 dpf larvae for *sbds^+/+^* and *sbds^nu132/nu132^*. The *sbds^nu132/nu132^* mutants possess a decreased number of neutrophils. Original magnification, ×6.3. (**I**) Presence of neutrophils at 15 dpf in *sbds^+/+^* and *sbds^nu132/nu132^* using the *Tg(mpx:Dendra2)^uw4^*. Original magnification, ×20. Number of neutrophils (**J**) at 5 and (**K**) at 15 dpf; *N* = 59 and *N* = 96, respectively. ANOVA test. *P < 0.05, **P < 0.001, ***P < 0.0001.

A major obstacle in understanding the pathophysiology of SDS and the role of SBDS in development has been the absence of an organismal model. Mice with ablation of SBDS die early in development (E6.5) (34). In contrast, our newly generated mutant zebrafish strains survive beyond embryonic and early larval stages. We evaluated mortality rates in both *sbds*-mutant lines (*sbds^nu132^* and *sbds^nu167^*). Both fish strains showed the expected Mendelian ratio of 1:2:1 during the first 15 dpf (Figure 2A and Supplemental Figure 2C). However, mortality rates increased for *sbds^nu132/nu132^* and *sbds^nu167/nu167^* at 21 dpf to 95%–97% (*P* = 0.0038 and *P* = 0.0173, respectively) (Figure 2A and Supplemental Figure 2C). Because SDS occurs in the compound heterozygous state, we intercrossed both fish strains and observed similar mortality rates for *sbds^nu132/nu167^* transheterozygotes (Supplemental Figure 2C).

To ensure that mutated *sbds* was causing the low survival rates in mutants, we created a transgenic line that expresses *sbds* ubiquitously. Our analysis showed a similar level of expression, which suggested a physiological level of expression of transgenic Sbds (Figure 2B). An elevated level of Sbds expression in *sbds^nu132/+^* and *sbds^+/+^* is not deleterious and did not produce a noticeable phenotype. Homozygous mutant fish carrying the transgene survived and were fertile (Figure 2C).

The hallmarks of SDS are short stature, neutropenia, and pancreatic exocrine insufficiency due to atrophy. Clinically, height and weight growth in children with SDS begin to fall off their trajectories before 2 years of age (37). To assess growth rates of larvae of different genotypes, we measured standard length (SL) at several time points. We observed a statistically significant reduction of SL in both mutant strains (*sbds^nu132/nu132^* and *sbds^nu167/nu167^*) at 15 dpf and 21 dpf but not in earlier stages (Figure 2, D and E). Similar results were found for compound heterozygous fish (data not shown). During regeneration, a massive cell reprograming and proliferation takes place. No significant differences in regenerative capacity were found at 15 dpf, despite reduced overall body size. In contrast, we observed significant reduction of the fin regeneration in *sbds* mutants at 21 dpf (Figure 2, F–H).

Neutropenia is found in 90% of SDS patients. To determine if it is present in *sbds* mutants, we crossed heterozygous zebrafish with transgenic reporter fish for neutrophils, *Tg(mpx:Dendra2)^uwm4^*, or macrophages, *Tg(mpeg1.1:Dendra2)^uwm12^*. Neutrophil numbers were significantly lower in *sbds^nu132/nu132^* compared with their WT or heterozygous siblings at 15 dpf (Figure 2, I–K; and Supplemental Figure 3A). However, no statistically significant differences were found in macrophage numbers (Supplemental Figure 3B). O-dianisidine staining for hemoglobin revealed no differences between *sbds^nu132/nu132^* and WT siblings at 5 or 10 dpf (Supplemental Figure 3C).

**Figure 3:**
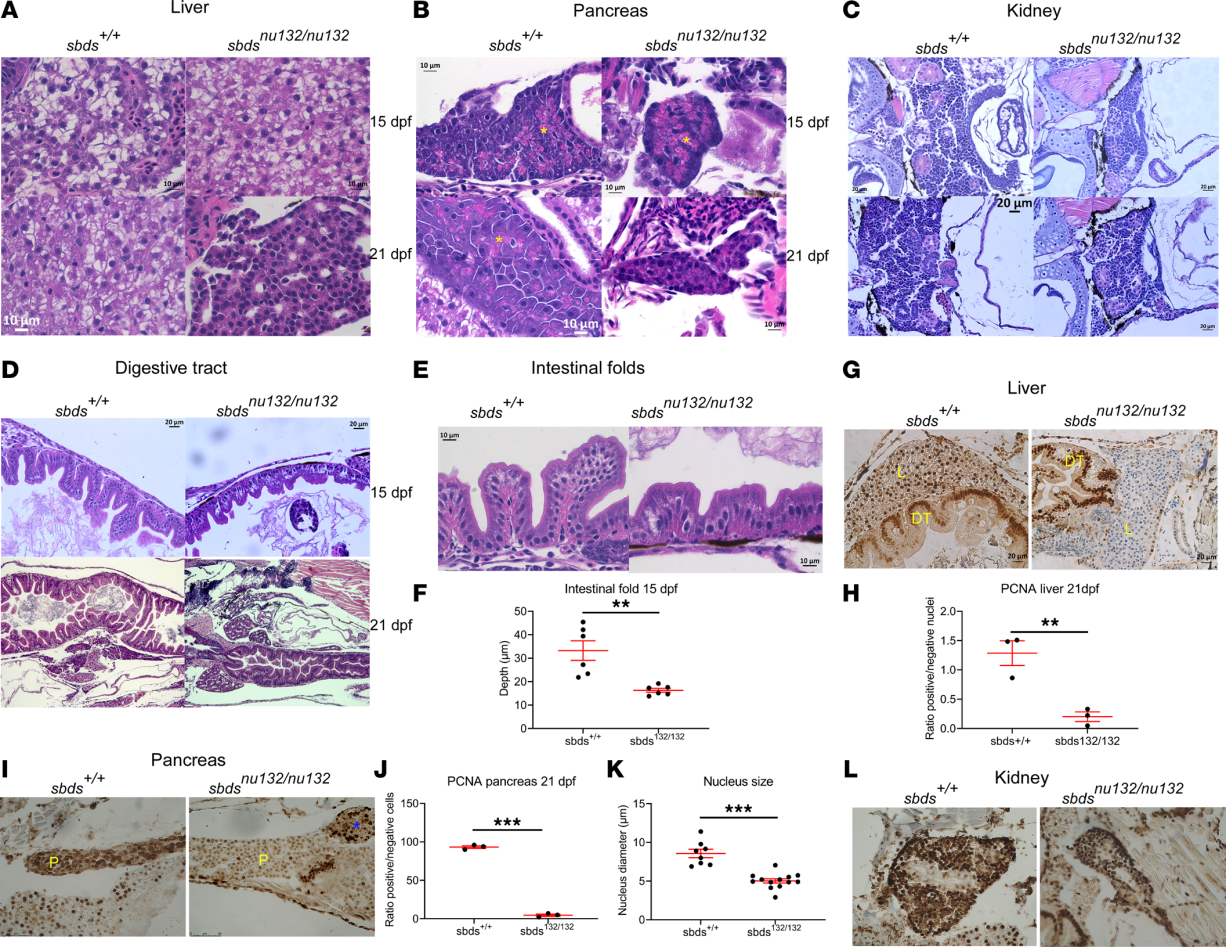
*Sbds^nu132/nu132^* mutants lead to defects in the liver, pancreas, and digestive tract. H&E staining of (**A**) liver, showing no differences between *sbds^+/+^* and *sbds^nu132/nu132^* at 15 dpf and liver fibrosis in *sbds* mutants at 21 dpf (original magnification, ×40); (**B**) pancreas, showing reduction of zymogen granules (yellow stars) (original magnification, ×40); (**C**) kidney and (**D**) digestive tract, showing a reduction in folds’ depth at 15 dpf and a constriction at 21 dpf in *sbds* mutants (original magnification, ×20); (**E**) intestinal folds at higher magnification (original magnification, ×40); and (**F**) quantitative differences in the depths of the epithelial folds. Immunohistochemistry for proliferation using PCNA in (**G**) liver at 21 dpf; (**H**) ratio of PCNA^+^ nuclei to total nuclei in the liver; (**I**) immunohistochemistry of pancreas, with blue star denoting the islet, at 21 dpf; and (**J**) ratio of PCNA^+^ nuclei to total nuclei in the pancreas and (**K**) kidney; no differences were detected in the ratios of positive nuclei to total nuclei (data not shown). (**L**) Quantification of the nucleus size in pancreatic acinar cells of *sbds* mutants versus WT siblings. *P < 0.05, **P < 0.001, ***P < 0.0001, *t* test. DT, digestive tract; L, liver; P, pancreas.

Histological examination was carried out to determine tissue architecture, presence of atrophy, and specific tissue involvement at different stages of development. No changes were found in major internal organs at 10 dpf (data not shown). Of particular relevance, the liver and pancreas did not show any structural differences at 15 dpf. However, by 21 dpf, the mutant liver demonstrated hepatocyte atrophy (average hepatocyte size at 21 dpf was 7.2 μm versus 11.95 μm at 15 dpf), which was accompanied by sinusoidal dilatation and overall reduced liver size (from average 619 μm in greatest dimension at 15 dpf to 316 μm at 21 dpf). The pancreas showed reduced greatest dimension on average versus the control group (245 μm in the study subjects versus 287 μm in the control subjects at 21 dpf), which raises the possibility of pancreatic lobular exocrine atrophy. In addition, the zymogen granules in pancreas were only observable at a greater magnification under the microscope in the mutant subjects at 15 dpf and at 21 dpf (noted at original magnification ×15–20 on the control group versus at original magnification ×40 on the mutant subjects), which may suggest that the zymogen granules are smaller and/or less developed in the mutant subjects (Figure 3, A and B). The site of blood cell production, the kidney, showed normal morphology in all stages studied (Figure 3C). The digestive tract showed a reduction of intestinal folds in *sbds* mutants at 15 dpf and was contracted at 21 dpf (Figure 3D). Intestinal fold depths were significantly shallower in mutants (Figure 3, E and F). The enterocytes revealed smaller greatest dimension at 21 dpf in the homozygous mutants when compared with the WT siblings (WT average greatest dimension 20 μm versus 12 μm on the mutant subjects). The *sbds^nu132/nu132^* genotype did not affect the fish’s ability to ingest food, as we observed a similar presence of food inside the intestine of *sbds* mutants and their WT siblings at 10 and 15 dpf (Supplemental Figure 4). To investigate the cell proliferation rate in different organs, we examined the expression of proliferating cell nuclear antigen (PCNA). Consistent with histological changes, we found significant reduction in cell proliferation in liver and pancreas at 21 dpf in *sbds* mutants (Figure 3, G–J). Nucleus diameter of acinar cells was significantly smaller in mutants (Figure 3K). No changes in proliferation were found in kidney (Figure 3L).

**Figure 4:**
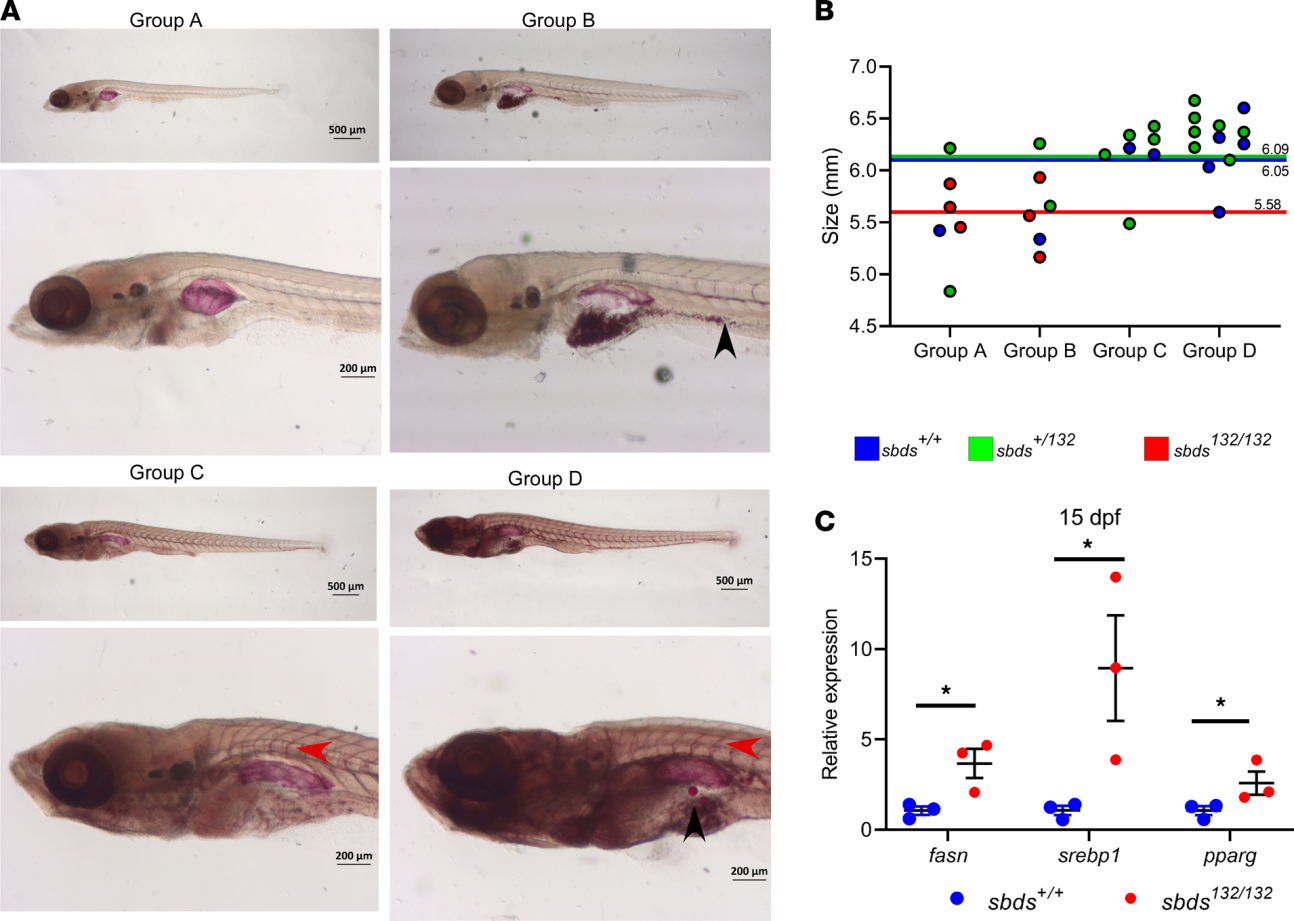
*Sbds^nu132/nu132^* mutants show a decrease in lipid accumulation. (**A**) Oil Red O (ORO) staining for neutral lipid accumulation: different groups depending on ORO staining. Red arrow shows lipid accumulation in the blood vessels; lipid droplets are indicated by a black arrow. (**B**) Size distribution and genotypes depending on the ORO staining. Colored lines show the mean SL for each group. (**C**) Gene expression of lipid metabolism markers in 15 dpf larvae. Statistical analysis was performed using the *t* test. *P < 0.05.

To investigate neutral lipid accumulation in mutants, 15 dpf larvae from an incross of *sbds^nu132/+^* were stained with the neutral lipid dye Oil Red O (ORO) and then compared according to their genotypes. Before genotyping, we created 4 groups depending on the staining: group A, pale with no ORO staining in the blood vessels; group B, pale but with small lipid droplets in the gut; group C, blood vessels strongly stained; and group D, blood vessels strongly stained with lipid droplets in the gut (Figure 4A). Fish were then assigned by a blinded reviewer to 1 of the 4 groups. Mutants were more likely to be found in the less stained groups (A and B) and were smaller than their WT and heterozygous siblings (Figure 4B). The reduction in lipid accumulation in mutants led us to interrogate how lipid metabolism was affected. We analyzed by RT-qPCR several key regulators: *fasn* (fatty acid synthase), *srebp1* (sterol regulatory element binding protein 1), and *pparg* (peroxisome proliferator activated receptor γ). We observed increased expression of all 3 at 15 dpf (Figure 4C).

### Transcriptional analysis identifies Tp53-associated gene upregulation.

**Figure 5:**
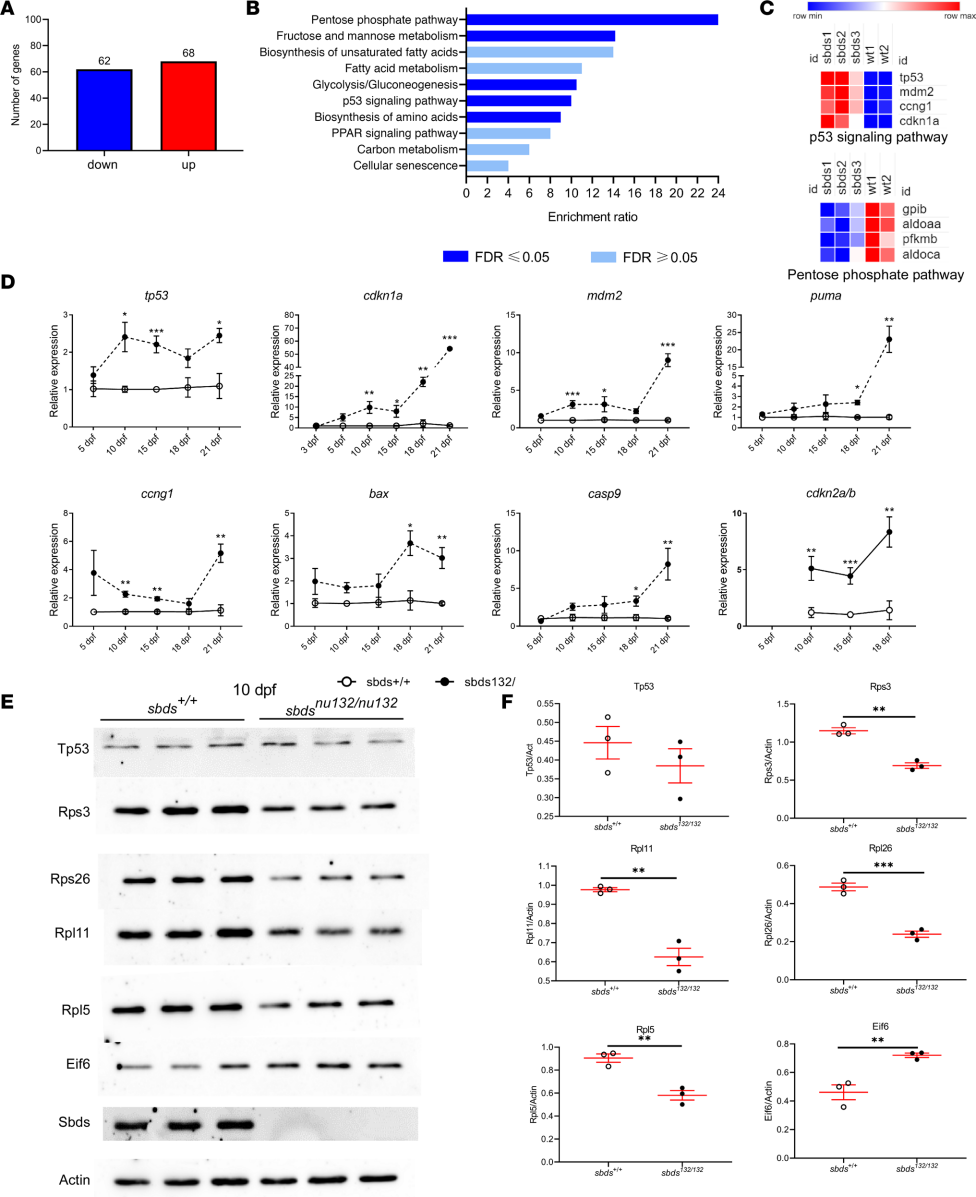
Transcriptional analysis identifies the upregulation of Tp53-associated genes, whereas Western blotting demonstrates a decrease in ribosomal proteins. **RNA-Seq results.** (**A**) Bioinformatic analysis of differentially expressed genes. (**B**) Gene enrichment analysis. (**C**) Heatmap of selected genes in Tp53 and pentose phosphate pathways. Relative expression based on fragments per kilobase of transcript per million mapped reads (FPKM) values significantly different between *sbds* mutants and WT. The color scale at the bottom represents the expression level, where red, blue, and white colors indicate upregulation, downregulation, and unaltered expression, respectively, on FPKM values. RT-qPCR analysis (**D**) mRNA levels at different time points of genes related to Tp53 pathway. (**E**) Western blotting at 10 dpf, 3 biological replicates for *sbds^+/+^* and *sbds^nu132/nu132^*. Two independent experiments with *N* = 3 each. (**F**) Quantification of Western blots using ImageJ (NIH). Statistical analysis was performed using the *t* test. *P < 0.05, **P < 0.001, ***P < 0.0001.

To determine possible mechanisms for the organ atrophy, we performed transcriptome analysis. RNA-Seq analysis of 15 dpf larvae revealed 68 upregulated and 62 downregulated genes in mutants (Figure 5, A–C). Consistent with our initial observations, *sbds* was downregulated in mutants (*P* < 5.85 × 10^–13^). Four genes related to the Tp53 pathway were upregulated: *tp53*, *cdkn1a* *(p21)*, *mdm2*, and *ccng1*. To validate the differences in gene expression obtained by RNA-Seq, we performed RT-qPCR at different developmental stages (3, 5, 10, 15, 18, and 21 dpf). Most of the genes studied showed significant overexpression in *sbds^nu132^* mutants at 10 dpf, which corresponds with the undetectable level of Sbds protein. A modest upregulation of *tp53* during 10, 18, and 21 dpf (1.5- to 2-fold) was observed. Similarly, *cdkn1a* expression was 3- to 5-fold upregulated at 10–18 dpf, and at 21 dpf it was 80 times higher than in the control. Targets of Tp53 involved in the apoptosis pathway also showed increased expression over time (*puma*, *mdm2*, *ccng1*, *cdkn2a/b*, *casp9*, *bax*) (Figure 5D). Similar profiles were found in *sbds^nu167^* mutants at 10 dpf (Supplemental Figure 2B). We also determined levels of Tp53, Eif6, and different ribosomal proteins (RPs; Rpl11, Rpl5, Rpl26, and Rps23) at 10 dpf by Western blotting (Figure 5, E and F). We found significant reduction in all 4 of the RPs. Tp53 protein levels were not significantly affected in mutants. Interestingly, a significant increase of Eif6 was found in mutants at 10 dpf and 15 dpf (Figure 5, E and F; and Supplemental Figure 2, D and E). This Eif6 increment was also found in *sbds^nu167/nu167^* mutants at 10 dpf (Figure 1C).

### Loss of tp53 partially suppresses sbds mutant lethality.

To test the hypothesis that the *sbds* mutant phenotype was Tp53 dependent, we generated *sbds^nu132/nu132^ p53^zdf1/zdf1^* double mutants by incrossing *sbds^nu132/+^ p53^zdf1/zdf1^* parents and analyzed the progeny at 4 months of age. No *sbds^nu132/nu132^* were found in over 70 fish. When we screened for the presence of mutants at 21 dpf, we found a frequency of 15.4% ± 2.9% of *sbds^nu132/nu132^* in 2 clutches (*N* = 30 and *N* = 45) (compared with historical levels of 1%–2%), but no mutants were found at 34 dpf (*N* = 56). Our results indicate that even when Tp53 is eliminated, the fish still required Sbds for survival into the juvenile and adult stages.

### Sbds mutants show starvation features.

**Figure 6:**
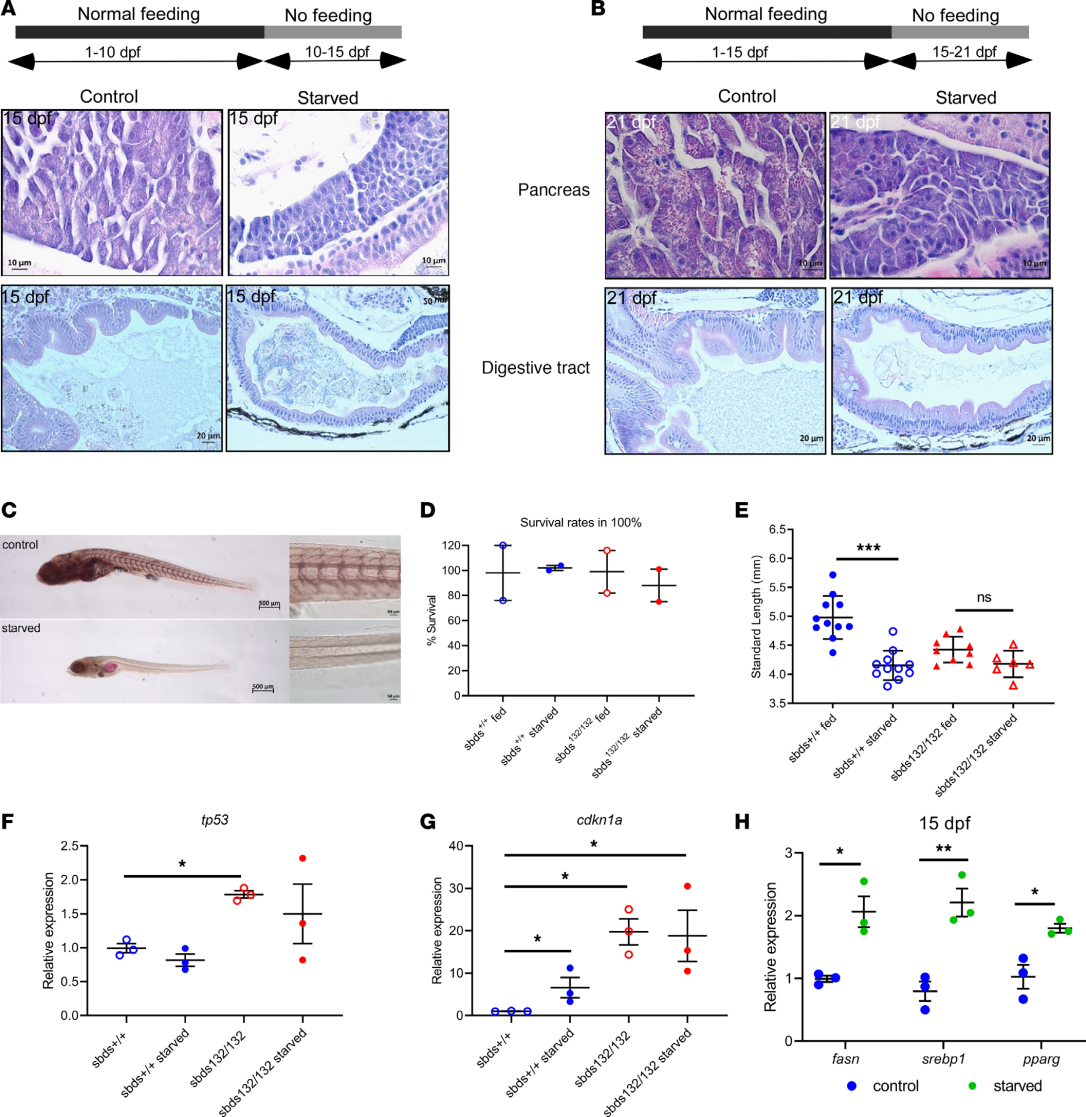
*Sbds* mutants show some features of starved fish. (**A** and **B**) H&E staining of pancreas and digestive tract in starvation assays. (**C**) ORO staining for lipid accumulation in starved and fed fish for 10–15 or 15–21 dpf. A model of starvation was performed to determine size distribution and fin regeneration. Scale bars: 500 μm (left), 50 μm (right). (**D**) Survival rates and (**E**) size distribution in WT and mutants after starvation. Expression of (**F**) *tp53* and (**G**) *cdk1a* show a dysregulation of *cdkn1a* in starved mutants. (**H**) Gene expression of lipid metabolism markers in starved WT fish. Statistical analysis was performed using the ANOVA and *t* test. *P < 0.05, **P < 0.001, ***P < 0.0001.

To study if the phenotype was due to starvation, we carried out 2 assays with WT fish. In the first assay we starved fish from 10 dpf to 15 dpf. We did not find differences in survival rates compared with the control group (Supplemental Figure 5A). Interestingly, the results in mutants demonstrated a reduction of zymogen granules in the pancreas and a reduction of the absorptive area in the digestive tract (Figure 6A). Similar results were found when we starved WT from 15 to 21 dpf (Figure 6B and Supplemental Figure 5B). ORO staining revealed a reduction in accumulated lipid in starved fish similar to that observed in *sbds* mutants (group A in Figure 4A) (Figure 6C). We also measured a significant reduction in size and fin regeneration capacity in starved fish compared with controls (Supplemental Figure 5, C and D).

When we carried out the starvation assay 10–15 dpf with progeny from an incross of *sbds^nu132/+^* fish, we found no changes in survival rates in WT or mutants in starved and control groups (Figure 6D). As we expected, we also found a significant reduction in size in WT fish starved compared with control WT nonstarved. However, starvation did not have a significant effect on the mutants’ size (Figure 6E). mRNA levels of *tp53* were not increased in starved controls, while *cdkn1a* levels were upregulated (5-fold). When we compared these levels in mutants, no changes were found. *Cdkn1a* was already upregulated in mutants (20-fold), and this level is very similar in starved mutants (Figure 6, F and G).

We investigated how starvation affected expression of RPs. We observed a decrease in all RPs tested (Rpl11, Rpl5, Rpl26, and Rps3) and Sbds (Supplemental Figure 5E). These findings are similar to those in *sbds* mutants. Interestingly, Eif6 showed no changes between starved fish and their fed siblings (data not shown). Moreover, we evaluated key regulators of lipid metabolism markers in starved fish. We found comparable changes in expression between starved (Figure 6H) and *sbds* mutants (Figure 4C).

## Discussion

SDS is the second most common cause of exocrine pancreatic insufficiency in childhood. The syndrome also classically involves blood and bone formation. An autosomal recessive disorder, it displays a variable phenotype that waxes and wanes. Almost half may not require pancreatic enzyme replacement, even though the pancreas has undergone fatty replacement, with deficient production of digestive enzymes (38). Other gastrointestinal defects involve intestinal atrophy, elevated transaminases, and hepatomegaly (39, 40). Nonalcoholic steatohepatitis and liver failure have been reported in at least 7 adults with SDS, with only 1 who had undergone stem cell transplantation (personal communication, Joan Mowery, Shwachman-Diamond Syndrome Foundation, Florence, Kentucky, USA) (41). Biallelic mutations involving *SBDS*, which encodes a ribosomal assembly factor, occurs in 90% of individuals. Other monogenic variants recently identified for SDS are *DNAJC21*, *EFL1*, and *SRP54*. All are involved in protein synthesis at the ribosome level (42). Fundamental gaps in our knowledge about the pathophysiology of SDS exist, in part due to paucity of organismal models.

*SBDS* is highly conserved among animals, plants, and archaea. In this study, we found that *sbds* is expressed at the 1-cell stage and during early embryonic stages in zebrafish. Using CRISPR/Cas9 editing, we reported 2 zebrafish *sbds* mutant lines, harboring a truncation or an insertion, that phenocopied the classic features of human SDS. Mendelian ratios, mortality, SL, and gene expression patterns showed in insertional mutants the same effects as in the deletional mutants. Unlike *Sbds^–/–^* mice, which die during early embryogenesis (34), the *sbds*-null zebrafish mutants survive through 21 dpf, with some reaching 4 weeks, corresponding to an early juvenile period. Furthermore, we identified a critical developmental period exists between 15 and 21 dpf when dysfunction and atrophy due to loss of Sbds occurred in multiple tissues and led to early mortality. Some of these effects were linked to increased expression of the Tp53-associated genes for growth arrest and apoptosis, but fish survival could not be rescued by loss of WT Tp53 protein. Moreover, specific gene targets affecting growth arrest and apoptosis were differentially regulated in magnitude of change and developmental stage.

We hypothesized that the mutated zebrafish survived because of the faster embryogenesis than mice. Our study showed a deposition of maternal mRNA and protein at the 1-cell stage that permitted the fish to survive during critical stages of embryonic development (e.g., high growth and proliferation). We found maternal Sbds protein through 5 dpf. During the cleavage stages of its early development, the embryo relies exclusively on maternally deposited RNA transcripts and protein, and cell cycles are rapid. At approximately 3 hours postfertilization, the embryo starts transcribing the zygotic genome at the maternal to zygotic transition, and cell cycles become longer (43). Because embryonic development in zebrafish is very fast, with all of the major organs developed by 5 dpf, the deposited maternal protein and mRNA likely overcame the critical stages of embryogenesis. These observations approximate the experimentally derived mammalian *SBDS* mRNA transcript half-life of 6 hours and protein half-life of 38 hours (44). In our zebrafish *sbds*-mutant strain, we were not able to determine whether truncated protein was present (because the epitope the antibodies recognized was at the C-terminus), but we detected downregulation of *sbds* mRNA by 3 dpf and onward. Protein stability of mutant SBDS is markedly reduced (45). Fish appearance and survival during embryogenesis were normal, likely due to the maternal deposition of Sbds. In the *sbds* morphant models, a more pronounced phenotype with lethality occurred by 5 dpf. This could be explained by morpholino-induced knockdown eliminating the translation of zygotic mRNA (35, 36). In addition, we could rescue the phenotype of the *sbds^nu132^* mutants by expressing the WT *sbds* ubiquitously, showing that the phenotype observed was due to the defect in Sbds.

**Figure 7:**
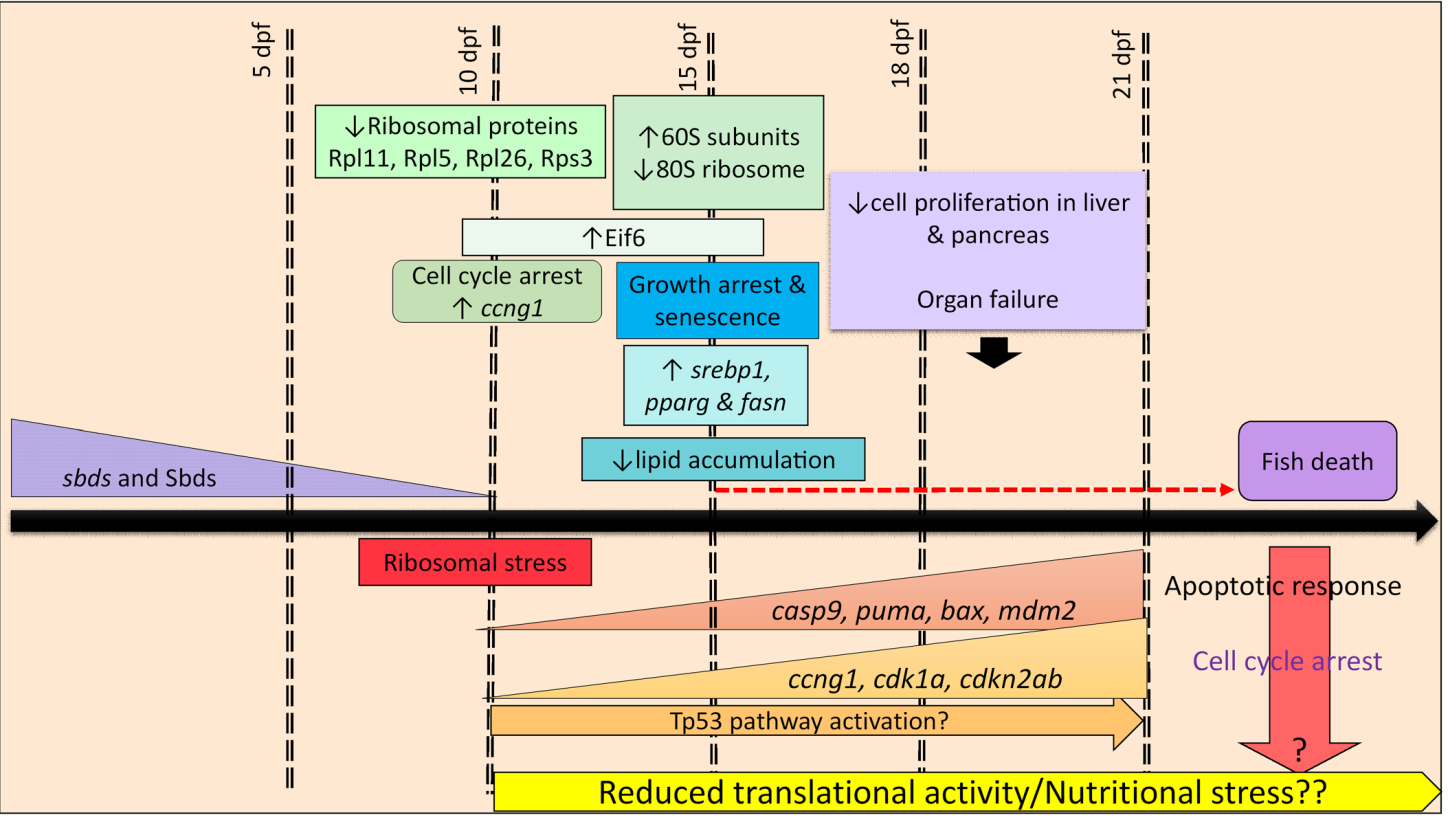
Scheme for the developmental pathophysiology of SDS based on phenotypic, biochemical, and genetic analysis of zebrafish mutants.

Even at this early developmental stage, our zebrafish model phenocopied the clinical aspects of SDS: neutropenia, smaller size, and pancreatic atrophy (Figure 7). Zebrafish provide a robust model for pancreatic development (46). Unlike Provost et al., who observed pancreatic hypoplasia in their *sbds* morphants (36), we did not observe any morphological differences in pancreatic size at 10 dpf using the transgenic line Tg(ptf1a:EGFPjh1) (data not shown). Additionally, the transcription factor Ptf1a is one of the earliest genes expressed in the developing pancreas, and its graded expression promotes development of both exocrine and endocrine pancreatic cells (47). Our RNA-Seq demonstrated equivalent levels of *ptf1a* expression in WT and sbds-deficient zebrafish even at 15 dpf (data not shown). Thus, pancreatic development is unhindered and atrophy occurs subsequently. In multiple organs, we were able to detect changes as early as 15 dpf, 7 to 10 days after Sbds protein levels disappeared. Defects in internal organs and mortality rates increased over the next 5 to 7 days. In addition, the zebrafish *sbds* mutants shared a number of characteristics with starved WT fish: smaller size, reduction in zymogen granules in the pancreas and shallower intestinal folds that causes a reduction of area of absorption for nutrients, reduction in accumulated lipids, upregulation of genes controlling lipid metabolism, decreased RPs, and reduced fin regeneration. We did not find a difference in size between fed mutants and starved mutants, which suggests physiologically significant malabsorption.

SBDS is required for the highly efficient assembly of 80S ribosomes (9). Homozygous mutations in 28 RPs in the zebrafish are embryonic lethal, while heterozygous fish are viable and fertile (48). Moreover, many of these heterozygous mutant RP fish display elevated cancer incidence (49). Patients with SDS, mutant mice, and yeast have lower levels of 80S, and that absence of SBDS provokes ribosomal stress (9, 10, 50–52). Polysome profile showed an increase of free 60S subunits and a reduction of 80S. Interestingly, we also found an increase of Eif6 levels, which play a role in the nucleocytoplasmic shuttling of the 60S subunit (50, 53).

Stress-induced Tp53 activation, such as from impaired ribosome biogenesis, constitutes a major cellular response mechanism. In response to stress, several RPs bind to MDM2 and block MDM2-mediated p53 ubiquitination and degradation, resulting in p53-dependent cell cycle arrest (54). Four of these proteins are RPL5, RPL11, RPS3, and RPL26 (55). RPL5 and RPL11 may also play a pivotal role in sensing stress resulting from an imbalance of RPs. Unexpectedly, Rpl5 and Rpl11 were downregulated in *sbds* mutants, while Tp53 protein levels were similar to their WT siblings at 10 and 15 dpf. Cell proliferation and architecture in the liver and pancreas were significantly decreased at 21 dpf (Figure 3, G–J), which might cause deficiencies in metabolism and nutritional stress, which could also lead to activation of Tp53-dependent and independent pathways.

Notably, Tp53 protein levels are not affected in mutants, and elimination of WT p53 in the *sbds* mutants did not rescue viability through the juvenile stage. Our fish strains provide a robust organismal model to understand Sbds function and stress response, tissue-specific gene targets in the pathophysiology and pathology of SDS. Tp53-independent pathways might include a specific nutritional deficiency, mitotic spindle dysfunction, or excessive TGF-β signaling (14, 56, 57).

## Methods

### Zebrafish husbandry.

Animals were maintained according to standard protocols at 28.5°C. In addition to new zebrafish lines described below, transgenic *Tg(mpx:Dendra2)^uwm4^* (58) and *Tg(mpeg1:Dendra2)^uwm12^* (59) and the mutant *tp53^zdf1^* line (also known as *tp53*^M214K^) (60) were used.

### Mutagenesis of zebrafish sbds.

CRISPR/Cas9 mutagenesis was based on the method of Gagnon et al. (61). Briefly, the web-based software “CHOPCHOP” (http://chopchop.cbu.uib.no) (62) was used to design a set of 2 sgRNA molecules (designated gRNA^XcmI and gRNA^BbslI, Supplemental Table 2) to target exon 2 of zebrafish *sbds*. sgRNA was transcribed in vitro using the pCS2-nCas9 (Addgene 47929) and MEGAshortscript-T7 kit (Ambion, Thermo Fisher Scientific). Cas9 was transcribed in vitro using the SP6 mMESSAGE mMACHINE kit (Ambion, Thermo Fisher Scientific). Both reagents were purified using columns (NucAway, Ambion, Thermo Fisher Scientific). sgRNA and Cas9 transcripts were coinjected into 1-cell–stage embryos. Each embryo was injected with approximately 2 nL of solution containing 12.5–25 ng/μL of sgRNA and 300 ng/μL of Cas9 mRNA in 0.3 M KCl (Supplemental Figure 1B). Genomic DNA was extracted from embryos at 1–2 days after injection for restriction site polymorphism-based genotyping (Supplemental Figure 1C). Primers are detailed in Supplemental Table 2. All obtained alleles were characterized by sequencing PCR products obtained from homozygous embryos.

### Zebrafish sbds rescue line.

To amplify *sbds* coding sequence, first the cDNA library was constructed using the SuperScript Master Mix VILO (Thermo Fisher Scientific) from total RNA isolated from adult zebrafish caudal fins (AB line from the zebrafish facilities at Northwestern University). Next, *sbds* was amplified using Q5 High-Fidelity DNA Polymerase (NEB) to add a *loxP* site in the 3′ end and subcloned in pDONR221 (Invitrogen, Thermo Fisher Scientific) by BP reaction. An inverse PCR reaction was carried out to remove GFP-*loxP* from the p5′-ubi:*loxP*-EGFP-*loxP* (63). We also used 3′ pE-GFP. Finally, a recombination reaction was carried out using these 3 plasmids and PDestTol2pA2 (64) to create a final construct ubi-*loxP*:sbds-pA-*loxP*:GFP.

### Polysome profile.

At 15 dpf larvae from an incross of *sbds^nu132/+^* were fin clipped and genotyped. Once the genotype was known, larvae were pooled in 3 groups, *sbds^+/+^*, *sbds^nu132/+^*, and *sbds^nu132/nu132^*, and treated with cycloheximide 100 μg/mL for 10 minutes, then sacrificed and stored at –80°C until lysis. Larvae were thawed and lysed at 4°C in polysome lysis buffer (10 mM Tris-HCl pH 7.4, 5 mM MgCl_2_, 100 mM KCl, 1% Triton X-100, 2 mM DTT, 100 μg/mL cycloheximide, complete EDTA-free protease inhibitor cocktail from Roche, and 500 U/mL RNasin Plus from Promega) by gentle trituration through a 26-gauge needle and incubation on ice for 30 minutes. The lysate was cleared of intact cells, nuclei, and mitochondria by centrifugation at 13,000 *g* for 10 minutes at 4°C. The supernatant was carefully layered onto an 11 mL 10%–50% linear sucrose gradient made in 20 mM HEPES pH 7.4, 5 mM MgCl_2_, 100 mM KCl, 2 mM DTT, and 100 μg/mL cycloheximide. Lysates were ultracentrifuged at 210,000*g* using an SW-41 Ti rotor (Beckman Coulter) at 4°C for 2.5 hours. Gradients were analyzed using a BioComp Piston Gradient Fractionator connected to a UV detector to monitor absorbance at 254 nm.

### RT-qPCR.

For WT zebrafish *sbds* analysis, pools of 20–30 embryos or larvae at different stages were collected. Larvae from incross between *sbds^nu132/+^* were collected at 3, 5, 10, 15, 18, and 21 dpf; fin clipped; and genotyped, and the body was kept in individual PCR tubes. Once the genotype was determined, pools of 3–7 larvae were used for RNA extraction (Supplemental Figure 6). We used at least 3 biological replicates in each experiment. Note that all 3 genotypes were from the same clutch, so all of them were siblings for each experiment. Larvae pools were homogenized, and RNA was isolated using TRIzol (Invitrogen, Thermo Fisher Scientific). cDNA was synthesized using the SuperScript Master Mix VILO (Thermo Fisher Scientific). Primers used are listed in Supplemental Table 3. Each experiment was performed in biological triplicates. mRNA expression in mutants relative to WTs was normalized to β-actin and calculated according to the ΔΔC_T_ method.

### Western blot analysis.

Protein was extracted from WT pools of 20–30 embryos/larvae at 1-cell stage; 50% epiloby; 1, 2, 3, 4, and 5 dpf; and zebrafish organs by using NP-40. Quantification was performed using BCA Protein Assay (Thermo Fisher Scientific). To identify homozygous mutants and their heterozygous and WT siblings, larvae were genotyped by fin clipping, and the rest of the body was kept in individual tubes and boiled for 3 minutes in Laemmli buffer with β-mercaptoethanol. Once the genotype was determined, samples were selected for Western blot and visualized with ECL prime (GE Healthcare Amersham). The following antibodies were used to detect: SBDS (Santa Cruz Biotechnology sc-271600), Tp53 (Anaspec 55342), EIF6 (NovusBio NBP2-16975), RPL11 (Cell Signaling Technology 18163), RPL5 (Cell Signaling Technology 14568), RPL26 (Cell Signaling Technology 5400) and RPS3 (Cell Signaling Technology 2579), β-tubulin (Cell Signaling Technology 2146), and β-actin (Santa Cruz Biotechnology sc-47778).

### Survival and SL.

Heterozygous fish for each *sbds* allele (*sbds^nu132^* and *sbds^nu167^*) were incrossed, and 50–80 descendants were imaged for SL as described in Parichy et al. (65) and genotyped at 3, 5, 10, 21, and 30 dpf. For compound heterozygotes we crossed *sbds^nu132/+^* and *sbds^nu167/+^* and genotyped their offspring at the same time points as described before.

### Histology.

Larvae at 10, 15, and 21 dpf were genotyped; euthanized; and fixed in 4% paraformaldehyde (PFA). Three larvae of each group were analyzed. H&E staining and immunohistochemistry with an antibody against PCNA (GTX124496, GeneTex) were performed in Northwestern University and Virginia Commonwealth University histology cores. For cell proliferation analysis, we counted PCNA^+^ nuclei in the pancreas and liver and calculated the total number of positive cells per nuclei using ImageJ (NIH). H&E-stained slides were reviewed by a liver pathologist and scanned using an Aperio scanner and software. Measurement of organs was performed using Aperio software and reported in micrometers. The H&E-stained slides were examined under an Olympus microscope.

### Detection of neutrophils and macrophages.

For neutrophil and macrophage detection we crossed *sbds^132/+^* mutants into the *Tg(mpx:Dendra2)^uwm4^* and *Tg(mpeg1.1:Dendra2)^uwm12^* transgenic lines, respectively. We crossed *sbds^nu132/+^*, analyzed the siblings at 5 and 15 dpf, performed live imaging, and then genotyped them. Neutrophils and macrophages were counted manually using ImageJ. For neutrophil detection we also used Sudan Black (21610, Electron Microscopy Sciences) as described previously (66).

### ORO staining.

At 15 dpf larvae were stained with ORO according to a protocol adapted from Schlombs et al. (67). Briefly, following anesthesia with tricaine (ANADA 200-226), zebrafish were fixed in 4% PFA at 4°C overnight and washed 3 times with 1× phosphate-buffered saline. Larvae were preincubated in 60% isopropanol for 30 minutes and dyed with 0.3% ORO for 3 hours. Samples were ready for microscopic observation following 3 washes with 60% isopropanol. After imaging, we measured the SL and then genotyped.

### o-Dianisidine staining.

As previously described (68), larvae were fixed in 4% PFA overnight; stained for 15 minutes in the dark in 0.6 mg/mL o-Dianisidine (MilliporeSigma), 0.01 M sodium acetate (pH 4.5), 0.65% H_2_O_2_, and 40% (*v/v*) ethanol; and were then bleached, imaged, and correlated with genotyping.

### Fin regeneration assay.

Fish were anesthetized with 0.4% tricaine, fin clipped under a stereoscope (ZEISS), and then put individually in a 24-well plate with 5 mL egg water. After 48 hours, fish were anesthetized, imaged, sacrificed, and genotyped as described above.

### Starvation assay.

Two starvation assays were carried out with WT fish: In starvation assay 1, fish were fed until 10 dpf and then starved until 15 dpf. In starvation assay 2, fish were fed until 15 dpf and then starved until 21 dpf. Control groups were fed normally in both assays. For mutants, we carried out starvation assay 1 using an incross of *sbds^nu132/+^*. These experiments were carried out with 50 controls and 50 starved fish each.

### RNA-Seq.

RNA was extracted from pools of 8–9 individually genotyped (Supplemental Figure 6) larvae at 15 dpf using TRIzol reagent. Three pools of *sbds* mutants and 2 pools of WT from the same clutch were compared. RNA quality was determined by Bioanalyzer (Agilent), and the *sbds* mRNA expression was measured by RT-qPCR. RNA-Seq library preparation and sequencing and mapping of 3 pools of *sbds^nu132/nu132^* and 2 *sbds^+/+^* were performed by the Beijing Genome Institute (BGI, Shenzhen, China). The library preparation followed the BGI’s standard procedure. Briefly, fragments were end-repaired, dA-tailed, adapter-ligated, and then amplified by a 4-cycle PCR program. The libraries were sequenced on the Illumina HiSeq 2500 platform using the 50 bp paired-end sequencing strategy (data set available at https://github.com/usuaoy/sbds-zebrafish-15-dpf#sbds-zebrafish-15-dpf). Based on the FPKM values (69), we used EBSeq R package for differentially expressed gene detection between *sbds^132/132^* and *sbds^+/+^* (fold change > 2, and *P* < 0.05). We performed a gene enrichment analysis for the genes under selection on each method using WebGestalt (70) and searching for Kyoto Encyclopedia of Genes and Genomes pathways. Heatmaps were made using Morpheus clustering web tool (https://software.broadinstitute.org/morpheus/).

### Imaging.

All images were taken using ZEISS stereoscopes (Stemi 508 and Discovery V8) and an AxioImager M2 microscope with a camera (Axiocam). Image analysis was carried out using ImageJ.

### Statistics.

Descriptive and analytical statistics were performed with Prism 6.0 (GraphPad Software). Parametric data are presented as mean ± SEM. The *n* values are indicated by dots in histograms; each individual *n* value represents a different animal. Statistical analysis used unpaired 2-tailed *t* tests or 1-way ANOVA with Tukey’s multiple-comparisons test. *P* < 0.05 was taken to indicate a significant difference.

### Study approval.

All zebrafish experiments were approved by the animal care usage committees at Northwestern University, Virginia Commonwealth University, and the Cleveland Clinic.

## Author contributions

UO, JT, and SJC designed the experiments; UO, WAM, MS, and MJK performed the experiments; DSA analyzed and reported on the gastrointestinal pathology; ANS and EC performed, interpreted, and wrote about the polysome profiling; UO, JT, and SJC analyzed and wrote the manuscript.

## Supplementary Material

Supplemental data

## Version 1 08/06/2020

In-Press Preview

## Version 2 09/03/2020

Electronic publication
