# Supplementary material for: Loss of Sbds in zebrafish leads to neutropenia and pancreas and liver atrophy
Source: JCI Insight. 2020 Sep 3;5(17):e134309. doi: 10.1172/jci.insight.134309 (PMC7526460; doi:10.1172/jci.insight.134309)
Supplement: Supplemental data [file jciinsight-5-134309-s056.pdf]

Table S1. *sbds* mutations found in F0

sbds mutations found in F0			
**Name**	**Nucleotide Sequence Change**	**Ins/Del**	**Effect**
sbdsnu129	189G>T+ 193G>A+195delGGCGAAG	Del	Truncation
sbdsnu130	190insC	Ins	Truncation
sbdsnu132	190delAAGTGGC	Del	Truncation
sbdsnu134	191delA	Del	Truncation
sbdsnu135	192delAGTGG + 199G>A	Del	Truncation
sbdsnu136	183delCAAGGGT	Del	Truncation
sbdsnu167	192G>A + 194insAGAAAGAAAGAAATGTGTCCA	Ins	Insertion 7 aa

Table S2. Primer for genotyping and sgRNA sequences

Primers for genotyping				
	**Forward (5'-->3')**	**Reverse (5'-->3')**	**Size (bp)**	**Enzyme**
sbds	TGACATGATTGGTTTAGGTTTG	CCCAGGTATATTATTTAGTCACAAGA	584	XcmI
sbds	TGATGAAGTCCTGCAAACCA	CAGGTCATCTGTTCCAAAAGC	100	
sbds	GCTTTGACCATTCAGATCACC	TCCTGCAAACCAACACAGTC	130	
p53	ACATGAAATTGCCAGAGTATGTGTC	TCGGATAGCCTAGTGCGAGC	336	MboII
**sgRNAs**				
sbds	sgRNA XcmI	GAATGTGTCCAAGGGTCAAG		
sbds	sgRNA BbsI	AAGACCTATCCAATGCTTTTG		

Table S3. RT-qPCR primer sequences

ZDB-GENE	Gene name	Gene	Forward (5'-->3')	Reverse (5'-->3')
ZDB-GENE-000329-1	Actin	*β-act*	TGCTGTTTTCCCCTCCATTG	TTCTGTCCCATGCCAACCA
ZDB-GENE-000511-6	Bcl2-associated X protein	*bax*	GGAGATGAGCTGGATGGAAA	AGATCTCACGGGCCACTCT
ZDB-GENE-030825-5	Caspase 9, apoptosis-related cysteine peptidase	*casp9*	CTGTCAAAGGGGGTCTTCAC	TCGTCTCCAGGTCTTTCACC
ZDB-GENE-020322-1	Cyclin G1	*ccng1*	CTTCTGTGCGGAGACGTTTT	ACAGCGATGTAGAAGCAGCA
ZDB-GENE-990415-153	E3 ubiquitin protein ligase	*mdm2*	TAACCGAGGCAGACTACTGGAAG	TTTCCCAGTTGGAGTGTGTTTCT
ZDB-GENE-070705-7	Cyclin Dependent Kinase Inhibitor 1A	*p21 (cdk1a)*	AGAGCTCGCGTGGAGTCAG	CCGAAAAGACTCCGCCTA
ZDB-GENE-070119-4	BCL2 binding component 3	*puma (bbc3)*	ACT GCC CCA CAT CCC CTC AC	CGT CCC CGA TTG TCC TCA GTT G
ZDB-GENE-040426-1116	Shwachman-Bodian-Diamond Syndrome	*sbds*	CCAACACAGTCTTTGTGAATG	CGCTTGGTCTCAGGATTC
ZDB-GENE-990415-270	Tumor protein p53	*tp53*	CTCTCCCACCAACATCCACT	ACGTCCACCACCATTTGAAC
ZDB-GENE-090812-3	Sterol regulatory element binding transcription factor 1	*srebp1 (srebf1)*	CTCTGGGTCACCGCTTCTTT	CAGATGCTCACGAAACCCCT
ZDB-GENE-030131-7802	Fatty acid synthase	*fasn*	GAGAAAGCTTGCCAAACAGG	GAGGGTCTTGCAGGAGACAG
ZDB-GENE-990415-213	Peroxisome proliferator-activated receptor gamma	*pparg*	CACTCTCCGCTGATATGGTGG	GTAGATGGGCTCGTGTGTCC

Figure S1

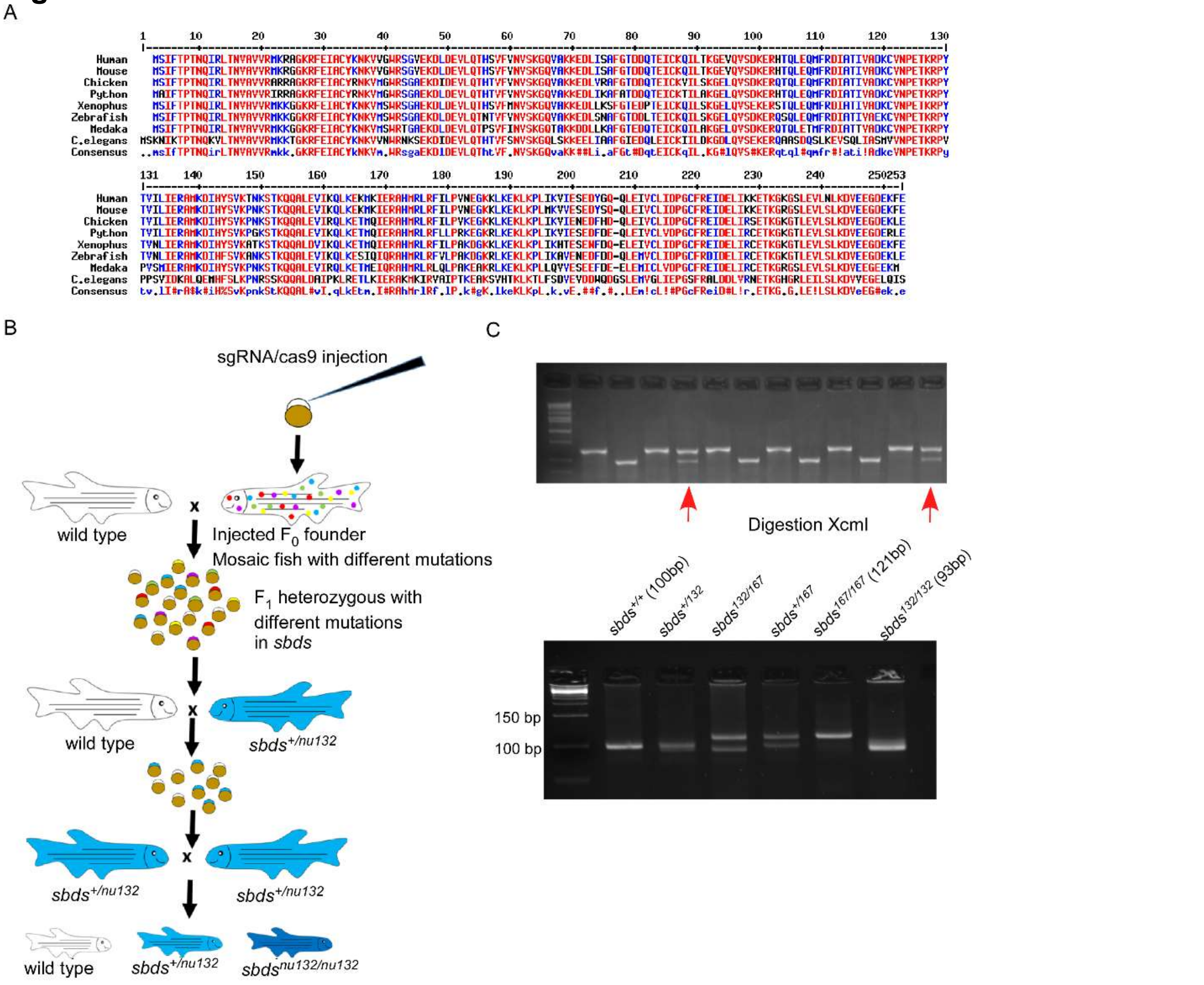

Figure S1. Generation and genotyping of zebrafish *sbds* mutants (A) Amino acid alignment of different species (alignment using Multalin). **(B)** Figure showing gRNA/cas9 injection and lines obtained. **(C)** Genotyping for the *sbds*$^{+}$, *sbds*^{nu132} and *sbds*^{nu167}.

Figure S2

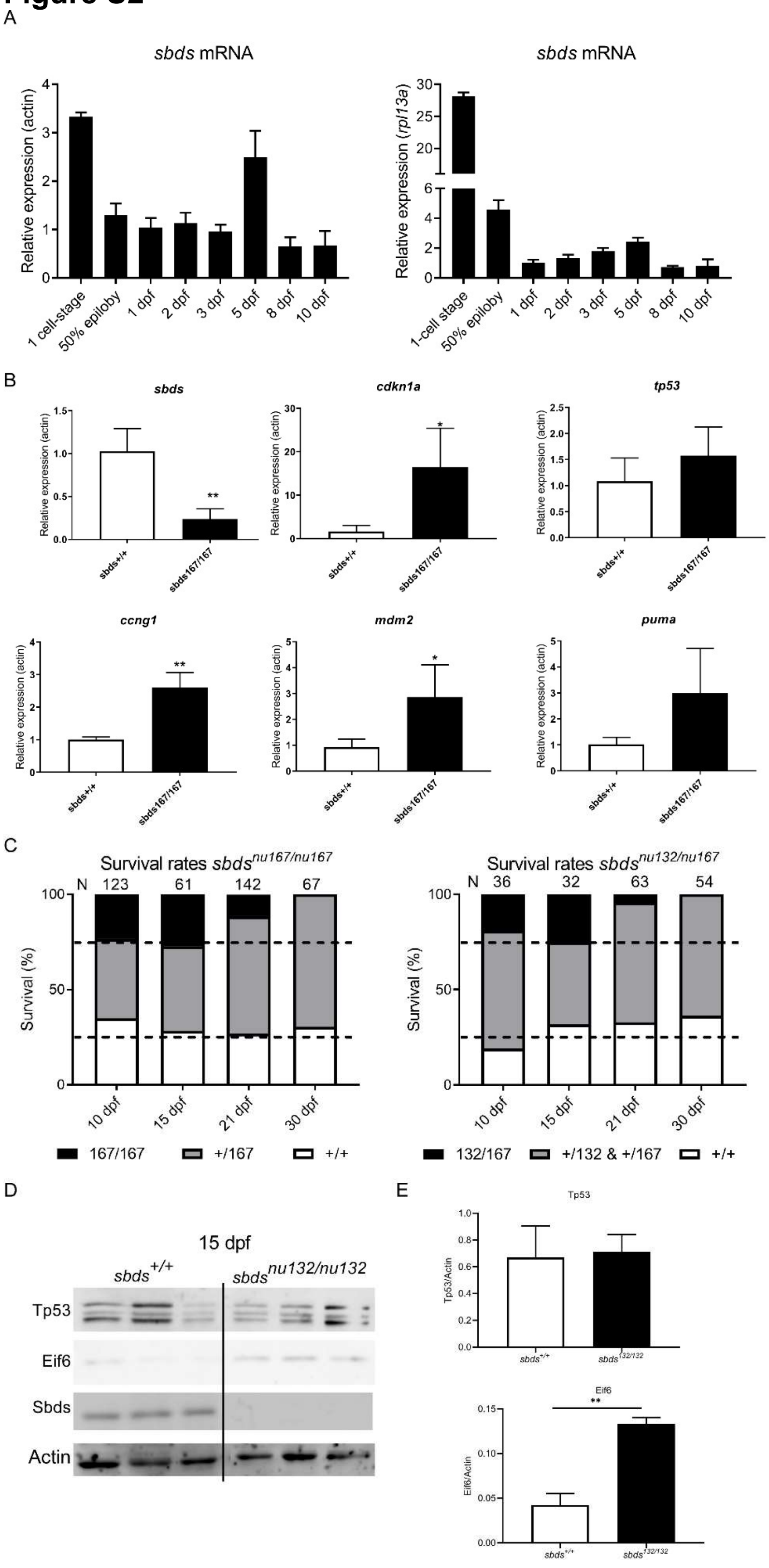
A
sbds mRNA
Relative expression (actin)
1 cell-stage
50% epiloby
1 dpf
2 dpf
3 dpf
5 dpf
8 dpf
10 dpf
sbds mRNA
Relative expression (rpl13a)
1-cell stage
B
sbds
cdkn1a
tp53
ccng1
mdm2
puma
Relative expression (actin)
sbds+/+
sbds167/167
C
Survival rates sbdsnu167/nu167
N 123 61 142 67
Survival (%)
10 dpf
15 dpf
21 dpf
30 dpf
167/167
+/167
+/+
Survival rates sbdsnu132/nu167
N 36 32 63 54
132/167
+/132 & +/167
+/+
D
15 dpf
sbds+/+
sbdsnu132/nu132
Tp53
Eif6
Sbds
Actin
E
Tp53
Tp53/Actin
sbds+/+
sbds132/132
Eif6
Eif6/Actin

Figure S2. **Transcriptional, protein, and survival analysis of mutant fish v. wild-type**. **(A)** *sbds* mRNA levels in wildtype zebrafish at different ages respect to two different housekeeping genes *b-actin* and *rpl13a.* Gene expression normalized to 1dpf. Pool 25-30 embryo/larvae and three biological replicates by stage. **(B)** mRNA levels at 10 dpf of *sbds*^{nu167}. **(C)** Survival rates for siblings of *sbds*^{nu167} and compound heterozygotes **(D)** Western blot and **(E)** quantification at 15 dpf of *sbds*^{nu132} allele* $p<0.05$: **$p<0.01$

Figure S3

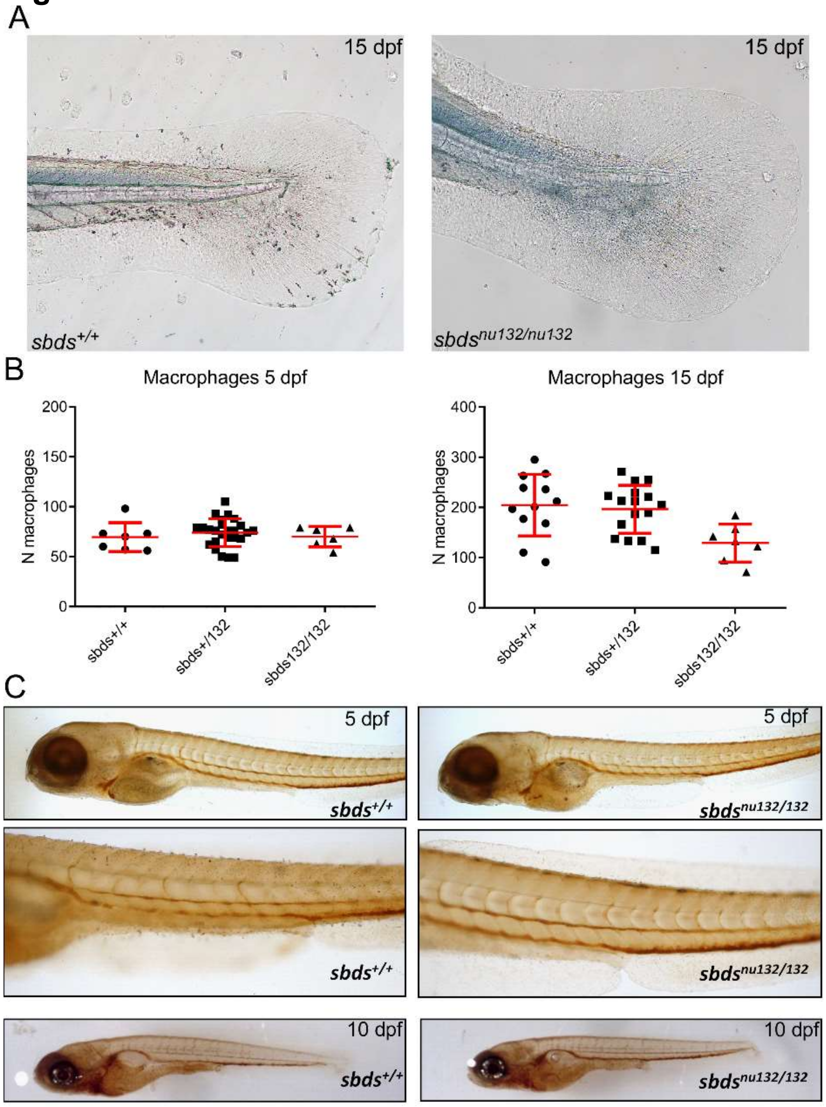

Figure S3. *sbds$^{nu132/nu132}$* mutants showed no changes in number of macrophages or hemoglobin appearance. (A) Sudan black staining for neutrophils at 15 dpf. **(B)** Number of macrophages at 5 and 15 dpf. The long horizontal lines denote the mean value. Statistical analysis was performed using ANOVA test. O-dianasidine staining for erythrocytes **(C)** at 5dpf and 10dpf**.**

Figure S4

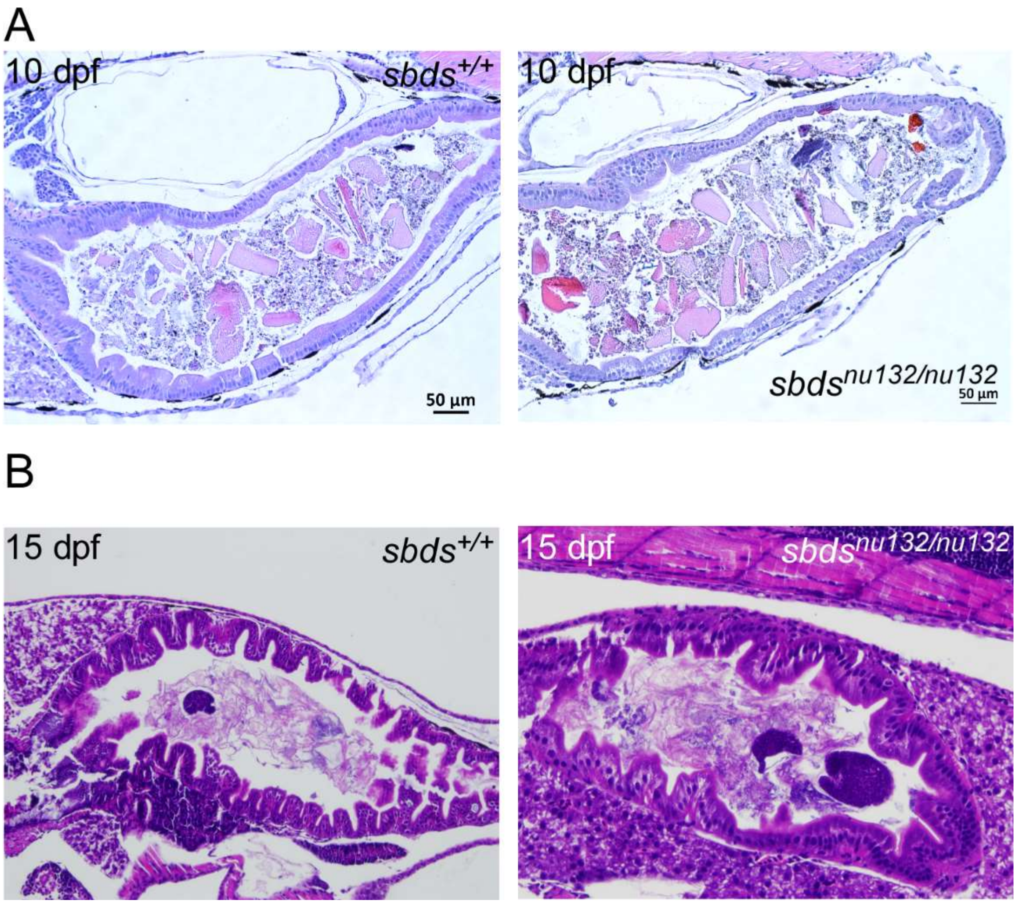

Figure S4. Comparable amounts of ingested food by *sbds* mutants and wild-type clutchmates. Histologic analysis of zebrafish intestine at **(A)** 10 dpf and **(B)** 15 dpf in mutants and siblings wildtype.

Figure S5

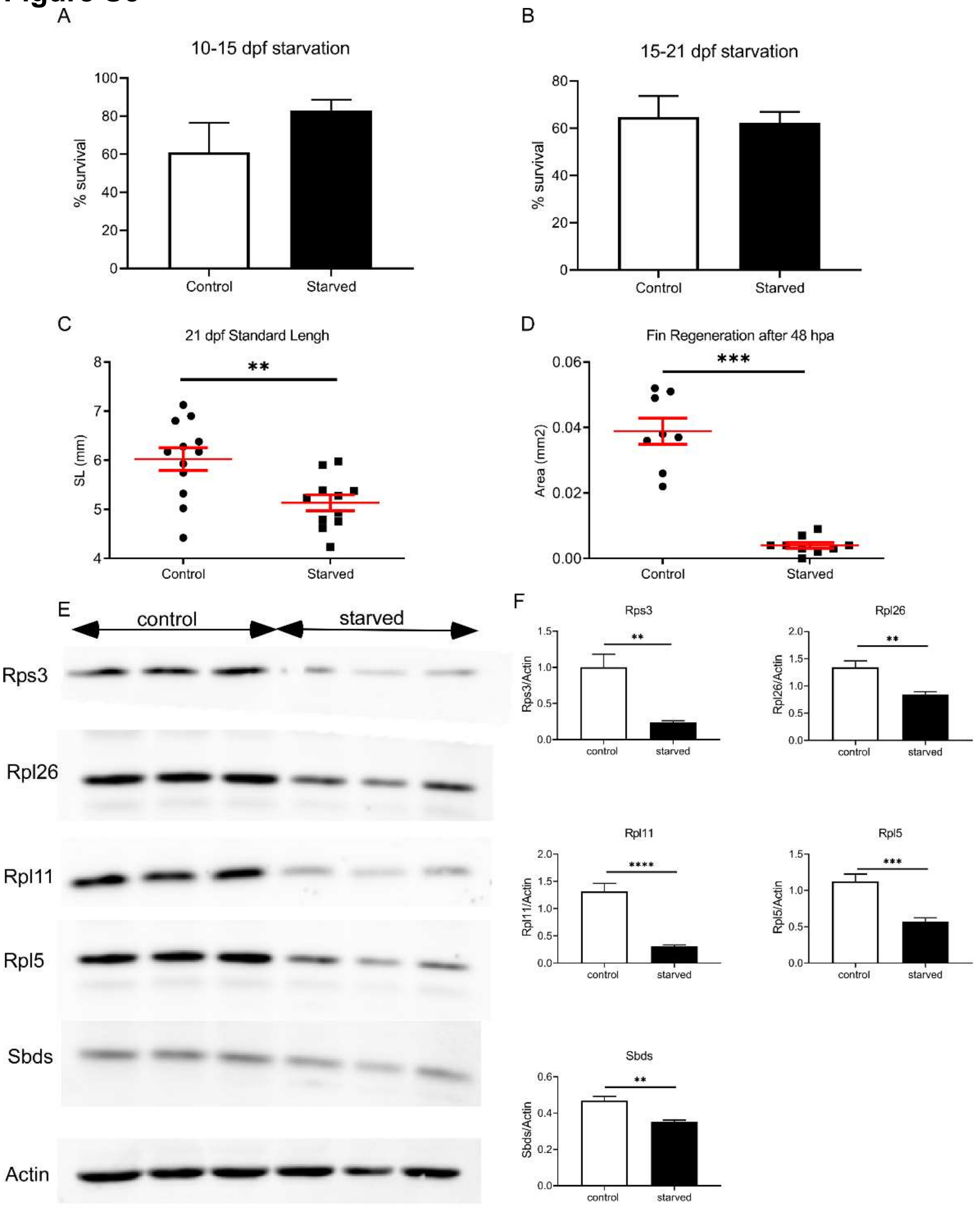

Figure S5. Phenotype of starved fish. Survival rates in wildtype fish after starvation from **(A)** 10 to 15 dpf and **(B)** 15 to 21 dpf. **(C)** Size distribution of starved fish and control group at 21 dpf. **(D)** Fin regeneration in wildtype starved fish is reduced compared to control group. **(E)** Western blot showing ribosomal proteins and Tp53 expression in 15 dpf larvae after 5 days of starvation. **(F)** Quantification of western blots. N=6

Figure S6

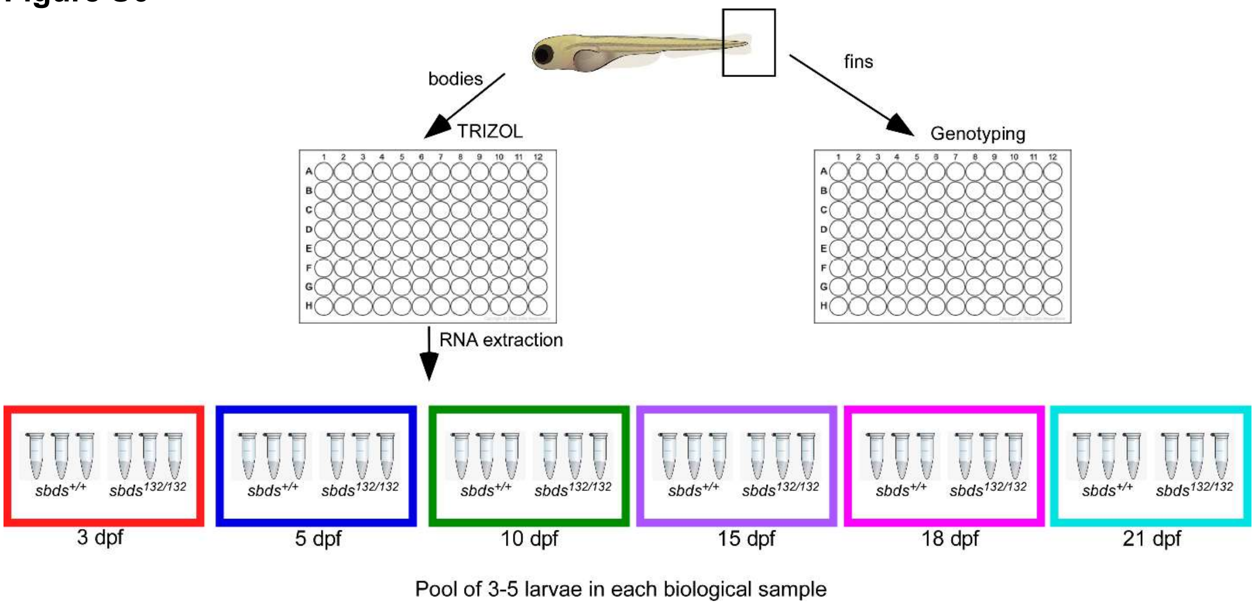

Figure S6. Schema showing the work flow for RNA extraction.
